# Neural and Nonneural Contributions to Wrist Rigidity in Parkinson's Disease: An Explorative Study Using the NeuroFlexor

**DOI:** 10.1155/2015/276182

**Published:** 2015-01-22

**Authors:** H. Zetterberg, G. E. Frykberg, J. Gäverth, P. G. Lindberg

**Affiliations:** ^1^Department of Neuroscience, Rehabilitation Medicine, Uppsala University, 75105 Uppsala, Sweden; ^2^Department of Women's and Children's Health, Karolinska Institutet, 17177 Stockholm, Sweden; ^3^Department of Clinical Sciences, Karolinska Institutet, Danderyd University Hospital, 18288 Stockholm, Sweden; ^4^FR3636 CNRS, Université Paris Descartes, Sorbonne Paris Cité, 45 rue des Saints-Pères, 75270 Paris Cedex 06, France

## Abstract

*Objective*. The NeuroFlexor is a novel method incorporating a biomechanical model for the measurement of neural and nonneural contributions to resistance induced by passive stretch. In this study, we used the NeuroFlexor method to explore components of passive movement resistance in the wrist and finger muscles in subjects with Parkinson's disease (PD). *Methods*. A cross-sectional comparison was performed in twenty-five subjects with PD with clinically identified rigidity and 14 controls. Neural (NC), elastic (EC), and viscous (VC) components of the resistance to passive extension of the wrist were calculated using the NeuroFlexor. Measurements were repeated during a contralateral activation maneuver. *Results*. PD subjects showed greater total resistance (*P* < 0.001) and NC (*P* = 0.002) compared to controls. EC and VC did not differ significantly between groups. Contralateral activation maneuver resulted in increased NC in the PD group but this increase was due to increased resting tension. Total resistance and NC correlated with clinical ratings of rigidity and with bradykinesia. *Conclusions*. The findings suggest that stretch induced reflex activity, but not nonneural resistance, is the major contributor to rigidity in wrist muscles in PD. The NeuroFlexor is a potentially valuable clinical and research tool for quantification of rigidity.

## 1. Introduction

Rigidity is one of cardinal features of Parkinson's disease (PD) [1]. Rigidity can be defined as an increased resistance to a passive movement and is considered constant throughout the range tested [1]. In diagnosed PD rigidity is known to fluctuate with treatment, that is, reduces with correct dose of treatment [2–4], and is therefore also an important measure of treatment response in clinical management. Clinically, rigidity is most commonly assessed as part of the motor section of the Unified Parkinson's disease rating scale (UPDRS). The examiner grades the overall rigidity according to severity, distribution (e.g., neck, arms, legs), and whether the rigidity is present at rest and in nonmedicated state. Despite extensive research the pathophysiology of rigidity remains unclear. For example, some research shows the involvement of velocity-dependent spinal stretch reflexes in rigidity [5–7], whereas it is traditionally considered to be independent of movement velocity [1]. Although the origin of rigidity is in the central neural pathways [2, 8, 9], some recent studies suggest that nonneural alterations in biomechanical properties of the stretched tissues (muscles, tendons, and connective tissue) may contribute to rigidity [10–13]. Increased elasticity in the stretched muscles has been found in PD subjects [11] and viscosity, reflecting velocity-dependent biomechanical resistance in stretched tissues, may also be increased and correlate with clinical rating of rigidity [10, 12].

Given its clinical importance and the diverse reliability of manual rigidity ratings [14–16] numerous methods have been proposed to objectively quantify rigidity in PD. These methods include surface electromyography [2, 6, 11], myometry [17], torque measuring devices [3, 5, 18–20], or manually imposed movement devices [10, 11, 14]. At present none of these methods measure the relative neural and nonneural contributions to rigidity. Nor have these methods been widely implemented in the clinical setting since they are often complex and time consuming.

The aim of this study was to explore the neural and nonneural components of passive movement resistance in the wrist and finger muscles in patients with PD using the NeuroFlexor method. The NeuroFlexor is a clinical method which measures passive movement resistance and quantifies its neural, elastic, and viscous components [21, 22]. According to recent findings we hypothesized that both the neural and nonneural components would be increased in PD subjects compared to age-matched healthy controls. We also set out to study the effects of activating the contralateral limb, a clinical approach used to increase rigidity, on the passive resistance components [23, 24]. We predicted that the contralateral hand activation maneuver would only affect the neural component since voluntary activation of the other limb is neural in origin. Finally we also examined how the measures of neural and nonneural resistance correlate to clinical scores of rigidity.

## 2. Methods

### 2.1. Participants

A total of 25 subjects with idiopathic PD participated in the study. Inclusion criteria for PD subjects were (i) diagnosis of idiopathic PD and (ii) presence of clinical sign of rigidity at the time of experiment. PD diagnosis was set according to (i) the presence of two of the three cardinal symptoms (hypokinesia, rigidity, and tremor) over the course of some months, (ii) the ruling out of other neurological disorders, and (iii) a favorable clinical response to levodopa treatment [1]. An age and gender-matched control group of 14 healthy nonneurologically impaired subjects also participated. Exclusion criteria for all participants were (i) an insufficient degree of passive wrist movement (<30° flexion and <40° extension); (ii) tension at rest in NeuroFlexor measurements (see Section 2.5); (iii) other neurological disorders, current disease or injury that affects arm and hand function; (iv) cognitive impairment that may affect ability to participate in the experiment. All PD subjects were on their ordinary medication, mostly with active substance levodopa, at the time of the experiment. The study was approved by the Regional Ethics Committee. All participants gave written informed consent in accordance with the Declaration of Helsinki before participating in the experiment. Details of the participants' clinical characteristics and medication are shown in Table 1.

### 2.2. Study Design

A cross-sectional comparison of chosen outcome measures between PD and control subjects was performed. Motor assessment according to the UPDRS was performed prior to NeuroFlexor measurements by the same rater to avoid being influenced by visualization of results. In the UPDRS protocol for assessment of clinical rigidity the selected arm is tested both passively (subject fully relaxed) and during a contralateral activation maneuver which increases the rigidity [2, 4, 23, 24], and this protocol was implemented in NeuroFlexor measurements, called passive and dynamic condition. The activation maneuver was ongoing in the contralateral arm during the NeuroFlexor induced stretch and consisted of fast rhythmic finger tapping or hand opening and closing.

### 2.3. Data Collection: Parkinsonian Rating Scales

Disease rating was assessed according to the Hoehn & Yahr scale and motor symptoms of PD were assessed using the UPDRS, part III (motor section) which consists of 14 items (maximum 108 points) [23].

### 2.4. Data Collection: The Biomechanical Model and NeuroFlexor Parameters

The biomechanical model has been previously presented and applied for the measurement of spasticity after stroke [21]. In the model the resisting force produced during passive wrist extension is regarded as a summation of passive elasticity, viscosity, and inertial forces, and by active muscle force. The model allows separate measurement of the passive movement resistance into* active* force produced by muscle contractions induced by stretch reflexes from the* passive* mechanical components. Although spasticity and rigidity differ in underlying mechanisms, rigidity has been shown to be associated with hyperactive stretch reflexes [2, 5, 6, 9] and should therefore result in increased neural contributions to passive movement resistance when measured using the NeuroFlexor. This is the first time that this model is applied in Parkinson's disease and we therefore briefly describe the model below.

**Figure 1:**
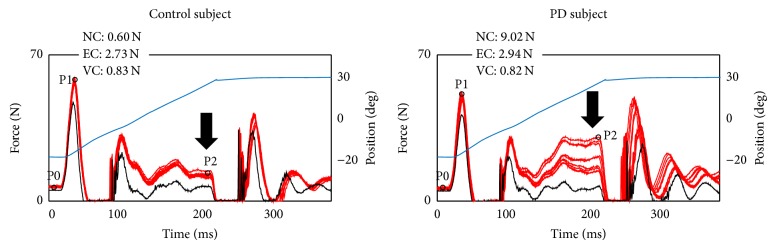
Example recordings in a control and a PD subject showing resistance profiles during high velocity movement (9 traces superimposed). Red traces show force recordings in Newton (N). Black trace shows resistance profile when the device runs empty (without hand). Blue trace shows degrees of movement. At the beginning and end of movement acceleration and decceleration forces are seen. The resistance increase during the constant phase of the movement (indicated by arrow) is crucial in determining the NC according to the biomechanical model. Three time points (P0, P1, P2) are automatically determined and the mean (across nine traces) is used in the software program for calculation of NC, EC, and VC. Values of each subject's calculated components are shown in the top left corner.

The NeuroFlexor software program was used to analyze resistance to stretch (in Newton) with specific time point values identified before stretch (P0), in the early phase of stretch (P1), and at the end of fast stretch (P2) (see Figure 1). For the slow trials force values were extracted at the end of the stretch. Points on the resistance profile are identified automatically for use in the model. These force points were used to estimate the* passive* (inertia, viscosity, elasticity) and* active* (neural) components.

#### 2.4.1. Inertia Component (IC)

Inertia is the force resisting the acceleration of the hand and depends on the mass of the hand and movable platform and the acceleration:

(1) $$\begin{array}{c} \text{IC}=m\times a, \end{array}$$

where IC is the inertia, *m* is the mass of hand and platform, and *a* is the acceleration.

The mass of the hand was estimated to be 0.6% of body weight [26].

#### 2.4.2. Elastic Component (EC)

Elasticity is a length-dependent resisting force that increases more the muscles and tendons are stretched [27]. In the model, the length-dependent elasticity is recorded 1 second after the end of the slow stretching movement (5°/s; P3), thus minimizing possible contribution from stretch reflexes.

#### 2.4.3. Viscous Component (VC)

The viscosity is the force produced by friction from neighboring particles, for example, sliding muscle fibers [28]. The viscosity depends on the velocity of the muscle stretch [27] and is highest during the initial acceleration and continues at a lower level during the remaining muscle stretch [27, 28]. In our model, the early viscosity component was defined as the resisting force that remained after the inertia component had been subtracted from the initial peak of the total resisting force at P1:

(2) $$\begin{array}{c} \text{V}{\text{C}}_{\text{P}1}={\text{Total}\;\text{force}}_{\text{P}1}-\text{IC}. \end{array}$$

Whereas the early viscosity could be calculated from the force trajectory, the later viscosity had to be approximated. In a previous report, Halaki et al. described that there is a rather stable relationship between the early and late viscosities, in which the late viscosity is about 20% of the early viscosity [29]. This relation was similar to that in stroke patients without visible EMG responses [21]. We therefore approximated the late viscosity at P2, to be 20% of the early viscosity at P1:

(3) $$\begin{array}{c} \text{VC}=\left({\text{Total}\;\text{force}}_{\text{P}1}-\text{IC}\right)\times 0.2. \end{array}$$

The late viscosity, at the end of the movement, was taken as the VC measure for each subject.

#### 2.4.4. Neural Component (NC)

The muscle stretch can activate a spinal stretch reflex with a latency of about 40 ms, followed by later stretch evoked responses adding to the first muscle contraction. In the model, the NC was estimated at P2 (maximal extension at the end of the passive movement) by subtracting the elasticity and viscosity components from the total force:

(4) $$\begin{array}{c} \text{NC}={\text{Total}\;\text{force}}_{\text{P}2}-\left(\text{EC}+\text{VC}\right). \end{array}$$

The NeuroFlexor (Aggero MedTech AB, Solna, Sweden) was used to quantify passive movement resistance during wrist extension and to calculate the various contributions according to the described model above. The method has been shown to be valid and reliable for the measurement of spasticity in stroke patients [21, 22]. Participants were seated comfortably with the instrument placed close to the side. A standardized position [21, 22] was used, with careful positioning of the hand in order to minimize measurement errors. The subject's shoulder was in approximately 45° abduction, the elbow in 90° flexion, and the forearm pronated. Subjects were instructed to relax the wrist muscles throughout the testing. Before the experiment started, subjects were given practice trials to become accustomed to the device. Range of wrist movement was 50°, starting position at 20° flexion and end position at 30° extension. Two velocities were used: slow 5°/s and fast 236°/s [21]. Five slow and ten fast stretches were performed during both the passive and dynamic condition. Subjects were randomized to a number of different sequences, to balance for order effects. The slow stretches were performed before the fast ones and there was a 10-second interval between each fast trial. The NeuroFlexor parameters measured included the neural (NC), elastic (EC), and viscous components (VC) of the total passive movement resistance.

### 2.5. Data Processing and Statistical Analysis

Data were organized in more versus less affected hand. In the PD group, the most affected hand was defined according to clinical rating of rigidity and hand motor function according to UPDRS. In the control group, the most affected hand was defined as the nondominant hand [30]. The first trials from slow and fast stretches were excluded from the analysis in order to avoid bias from startle reflexes and mechanical hysteresis [21]. NC, VC, and EC values in Newton were noted for each fast stretch (nine), which was analyzed in set with the remaining four slow stretches. For analysis of resting tension P0 values, reflecting force applied before onset of stretch, were noted for each trial (Figure 1). The total resistance was also calculated (= NC + EC + VC) for each trial. Statistical analyses were performed using Statistica 10 (StatSoft Inc., Tulsa, OK, USA). NeuroFlexor data was not normally distributed (Shapiro-Wilk test *P* < 0.05) and was therefore log transformed using the natural logarithm in order to perform repeated measures ANOVA. Before log transforming a constant of 3 N was added to all values to avoid negative and zero values [22]. The log transformed data was normally distributed (Shapiro-Wilk test *P* > 0.05) and the groups showed similar variance (Levene's homogeneity of variance test, *P* > 0.1). A repeated measures ANOVA showed no effect of repetition across NeuroFlexor measurements and a mean value across repeated trials was used in group comparisons. No differences were found between groups regarding resting tension before stretch (P0 values), during slow movement or at rest. To detect differences in NeuroFlexor variables between the PD group and control group repeated measures ANOVA was used, with two within-group factors (HAND, PASS/DYN) and one between-group factor (GROUP). Post hoc comparisons, using Fisher Least Significant Difference (LSD) test, were used to evaluate significant differences between factors. For investigation of correlation with clinical measurements Spearman's Rank Order Correlation coefficient was used. The UPDRS III score along with rigidity (UPDRS item 22) and bradykinesia (UPDRS item 24) was correlated with NeuroFlexor measurements. Log transformed values of NC, EC, and VC were used in correlation analysis. Correlation analyses were limited to NeuroFlexor variables showing group differences, to limit multiple comparisons. The level of significance was set to *P* < 0.05 for group comparisons and *P* ≤ 0.008 in correlation analyses (Bonferroni correction).

## 3. Results

### 3.1. Features of the Enrolled Patients

Mean age in PD group was 72 ± 5.9 (SD) years, mean time since diagnosis was 7 ± 5.3 (SD) years, and mean age for controls was 73 ± 4.9 (SD) years. There was no difference between PD and control groups regarding gender (*P* = 0.47) or age (*P* = 0.76). Motor function according to UPDRS rating varied widely (range 2–49; max 108) among participants as seen in Table 1. The PD subjects showed stable motor status and were in ON state during measurements. All NeuroFlexor measurements were successfully performed and none of the participants reported any discomfort during testing.

### 3.2. Factors Contributing to Passive Movement Resistance

In general, PD subjects showed increased passive stretch resistance compared to control subjects (see examples in Figure 1). For details regarding total resistance, components, groups, and hands, see Table 2. The largest contributor to resistance in PD subjects was the NC, whereas in the controls was mainly the EC. As regards the least affected hand, PD showed increased total resistance and NC in dynamic condition compared to controls (Table 2). As regards the most affected hand, PD showed increased total resistance and NC in both passive and dynamic conditions compared to controls (Table 2). Although no significant difference in the components was found between the most and least affected hands within groups, higher NC values were present in the most affected hand in 16 (64%) PD subjects. This was consistent with higher clinically rated rigidity in the most affected hand, which was present in 17 (68%) PD subjects (Table 1).

### 3.3. Contralateral Activation Maneuver

The total passive movement resistance was higher in both groups in the dynamic (i.e., with contralateral hand activation maneuver) compared to the passive condition. The NC and VC increased in the dynamic condition, whereas the EC decreased. The most prominent effect was in the NC showing an average increase in PD by 118%. The dynamic effect was smaller in VC increasing by 11% and EC decreasing by 7% in PD. As regards the least affected hand, the ANOVA analysis showed that PD had increased total resistance, NC, and VC in dynamic compared to passive condition (*P* < 0.006). PD also showed reduced EC in the least affected hand during dynamic condition (*P* = 0.01). As regards the most affected hand, PD showed increased total resistance and VC in dynamic compared to passive condition (*P* < 0.004). No other significant differences were identified. We analyzed the dynamic effect on resting tension (P0 values) by using ANOVA. This analysis showed increased P0 values in the dynamic condition compared to the passive condition for both PD and controls (PASS/DYN: *F*(1,35) = 10.5, *P* = 0.003, *η*_*P*_^2^ = 0.23). The statistical analyses (ANOVAs) were redone with P0 included as a covariate, to control for potential confounding effect of resting tension. Including P0 as covariate eliminated PASS/DYN ANOVA affects both NC and EC. Thus contralateral activation likely affects the NC and EC through an increase in baseline muscle activation before stretch.

### 3.4. Correlations between NeuroFlexor and UPDRS, Rigidity, and Bradykinesia

As regards the least affected hand, rigidity correlated with total resistance and NC in both the passive and dynamic conditions (Table 3). As regards the most affected hand, bradykinesia correlated with NC in the passive condition (Table 3). Rigidity in the most affected hand tended to be higher in patients with higher total resistance and NC in the dynamic condition, although not significant (Table 3). Both total resistance and NC showed nonsignificant tendencies to be higher in patients with poorer general motor scores according to UPDRS (part III).

## 4. Discussion

Our study demonstrates that the NeuroFlexor method is able to detect rigidity in PD subjects. As predicted, the biomechanical model identified an increased neural component of passive movement resistance in hand and finger flexor muscles in PD subjects, whereas the elastic and viscous components were similar to controls. Thus, using the NeuroFlexor the neural component for PD subjects was found to be the major contributor to passive movement resistance, whereas for controls it was shown to be the elastic component. The activation maneuver increased the NC in the PD group.

The results support that the neural component is the major contributor of rigidity in PD, with a stretch reflex induced increase in passive movement resistance. In contrast to previous studies [10–12, 31], we found no increase in elastic and viscous components in PD subjects. Hence nonneural resistance, quantified using the NeuroFlexor, does not seem to contribute significantly to clinically rated rigidity.

The involvement of stretch reflex activity in rigidity in PD subjects has been shown in some other studies [2, 5, 6, 9]. In this study we used slow and high velocity (5°/s and 236°/s) stretches to separate out the neural component according to a novel biomechanical model not previously applied in PD subjects. This method has been shown to trigger stretch reflex induced muscle resistance in stroke patients with spasticity [21]. The NeuroFlexor method does not differentiate between spinal and supraspinal stretch reflex activity, but earlier research suggests that only the supraspinal reflex loop is disturbed in rigidity [8, 9], as opposed to spasticity where both spinal and supraspinal pathways might be affected [32]. There is growing evidence for velocity dependence in rigidity [4–6, 20], which also supports increased activation of the stretch reflex. Animal models and fMRI studies suggest that subcortical, primary, and premotor cortical regions are involved in rigidity [33, 34].

The increased NC during the dynamic contralateral hand activation maneuver confirms previous findings [4], even though they used a static contralateral grip contraction. The physiological overflow effect (effect of muscle contraction in another body part) has been shown to influence strength [35] and H-reflexes [36] in healthy subjects. Cortical, spinal, and peripheral mechanisms have been suggested to mediate the crossed effects [35, 37, 38]. The findings of Powell et al. [4] that medication reduced the effect of activation maneuver in PD supports the hypothesis that cortical components mediate the crossed effects seen on muscle tension [37, 38]. The widespread cortical and subcortical networks suggested to be involved in rigidity [34] might explain the effect of contralateral activation maneuver, as well as the pronounced rigidity seen in other activities such as varied postures or attention [24]. The effect of contralateral activation was eliminated when controlling for resting tension before the muscle stretch. This suggests that the resting state of the muscle can explain the increase found in the dynamic condition. PD subjects may have difficulty in maintaining a relaxed state in muscles not used in a task and this is why rigidity increases during the contralateral activation maneuver. Interestingly, in the least affected hand the group difference only became apparent during the dynamic condition (no difference present in passive condition, Table 2). This agrees with the clinical observation that contralateral activation is useful in detecting presence of mild rigidity.

The correlation analyses showed that PD subjects with high NC had high clinical ratings of rigidity and bradykinesia. Correlations in the most affected hand did not reach significance (when corrected for multiple comparisons) which may be due to the reduced spread of rigidity values (0–3 in the least affected hand versus 1–3 in the most affected hand). Rigidity correlation coefficients were relatively similar to what has been found in previous studies using other techniques to quantify resistance profiles [6, 10, 12]. Higher correlations were noted in the condition with activation maneuver, which corresponds with clinical observations and rigidity correlated slightly better with total resistance compared to NC. This makes sense given that the clinician evaluates the total resistance to passive movement. The coherence with clinical findings suggests that the NeuroFlexor measurements (in particular NC) may be a useful marker of rigidity. However, more research is needed to establish the validity and reliability of the NeuroFlexor measures in PD and to confirm relations. Rigidity and bradykinesia have been shown to be related with synchronized oscillatory activity in the beta band in the subthalamic nucleus and dopaminergic medication reduces both rigidity and bradykinesia and this oscillatory activity [39]. In this study, NC correlated with both rigidity and bradykinesia, in line with similar mechanisms underlying these clinical signs. Longitudinal quantitative investigations of rigidity and its relationship with functional clinical outcome measures are also important future research topics.

Limitations of this study include the lack of EMG measurements, which could have validated study findings. Since this was a preliminary investigation measurements were not obtained during both on and off medication. One challenge when using the NeuroFlexor method is for the patient to remain fully relaxed during the passive stretches. This may complicate measurements in certain subjects with PD, for example, those with dystonia and hyperkinetic movements. Most likely the enhanced stretch reflex activity in PD is not specific of rigidity, but also present in dystonia and hyperkinesia. Hyperkinetic movements and pronounced tremor could also cause artefacts in mechanical assessment of the affected limb. In this study we thoroughly controlled for movement artefacts and found no differences between PD and controls in terms of tension at rest. Finally, using the NeuroFlexor we were not able to estimate the contribution of the shortening reaction [4, 40], where the resulting force would be in the direction of movement.

## 5. Conclusions

This study highlights the role of neural resistance as the major contributor to Parkinsonian rigidity. Nonneural properties (elasticity and viscosity) were only marginally increased in PD subjects. The increase and stronger correlation to clinically rated rigidity during contralateral activation suggest that eliciting neural crossed effects (interference from contralateral activated neural pathways) may increase measurement sensitivity. Further validation of the NeuroFlexor method for the quantification of resistance profiles in rigidity is warranted.

## Figures and Tables

**Table 1:** Participants' characteristics and medication.

PD subject	Age (years)	Gender	Disease duration (years)		UPDRS		UPDRS		NeuroFlexor		LED
				Hoehn and Yahr scale (1)	Part III score (2)	MA arm	Rigidity MA (3)	Rigidity LA	TOT MA (N) (4)	TOT LA (N)	
1	69	M	7	2	30	R	2	1	6.4	7.0	400
2	73	F	2	2	27	L	1	1	6.0	8.5	500
3	72	F	4	4	49	L	2	2	13.7	11.4	1605
4	66	F	1	2	16	R	1	1	7.9	7.3	200
5	72	F	1	1	2	R	1	0	10.7	7.6	300
6	63	F	1	1	5	R	1	0	5.8	4.6	600
7	67	F	7	2	13	L	1	1	8.8	9.5	823
8	71	M	4	3	46	L	3	1	16.1	7.1	599
9	79	M	2	1	11	R	1	0	6.8	5.1	300
10	81	M	5	1	19	R	2	0	13.6	4.1	200
11	77	M	3	2	21	R	2	1	8.0	5.6	500
12	73	F	7	4	45	L	2	2	13.1	19.6	1475
13	69	M	3	1	13	R	2	0	8.1	6.4	575
14	78	M	8	3	41	L	3	3	7.2	11.7	575
15	77	M	5	3	39	L	2	2	18.1	30.1	1000
16	66	M	11	3	40	L	3	2	14.2	14.9	1651
17	72	F	18	3	24	L	2	1	3.3	4.6	873
18	56	M	20	2	43	L	3	2	21.3	26.1	1075
19	73	M	18	2	15	L	3	2	10.3	10.2	733
20	76	M	9	2	8	R	1	0	6.9	5.2	1149
21	73	M	13	3	33	R	3	2	21.3	12.4	798
22	78	F	5	3	25	L	2	1	9.8	7.5	480
23	80	M	6	3	28	R	1	0	8.6	5.1	775
24	79	M	10	3	45	R	2	2	24.1	5.9	900
25	67	M	5	2	17	R	2	1	5.8	5.5	557

Mean ± SD	72.3 ± 6.0		7.0 ± 5.4	2 ± 1	26 ± 14				11.0 ± 5.5	9.7 ± 6.6	746 ± 404
		M: 16 F: 9				R: 13 L: 12	Median: 2	Median: 1			

PD: Parkinson's disease, M: male, F: female, R: right, L: left, MA: most affected, LA: least affected, N: Newton, and LED: levodopa equivalent dose [25].

(1) Disease rating 1–5 according to Hoehn and Yahr scale.

(2) Unified Parkinson's disease rating scale, motor section, maximum 108 p.

(3) Rigidity according to UPDRS: 0 = absent; 1 = slight or detectable only when activated by mirror or other movements; 2 = mild to moderate; 3 = marked; 4 = severe.

(4) Total resistance in NeuroFlexor measurements, mean of passive and dynamic condition.

**Table 2:** Mean values (±SD) of passive movement resistance for least and most affected hand in PD and control group during passive and dynamic conditions.

PD *n* = 25	Least affected hand						Most affected hand					
Controls *n* = 14	Passive			Dynamic			Passive			Dynamic		
	Control	PD	*P* value	Control	PD	*P* value	Control	PD	*P* value	Control	PD	*P* value
Total (N)	4.6 ± 2.6	7.5 ± 4.8	0.31	5.4 ± 2.4	11.6 ± 9.4	0.01	3.9 ± 1	9.7 ± 5.6	0.01	5.3 ± 4.7	11.9 ± 6.6	0.004
NC (N)	0.4 ± 1.9	2.3 ± 4.1	0.47	1.2 ± 2.3	6.6 ± 9	0.02	0.0 ± 1.1	4.8 ± 4.7	0.02	1.5 ± 4.5	7 ± 6.3	0.01
VC (N)	0.8 ± 0.4	0.9 ± 0.4	0.61	0.7 ± 0.3	1 ± 0.5	0.15	0.8 ± 0.4	0.9 ± 0.5	0.70	0.9 ± 0.5	1 ± 0.5	0.47
EC (N)	3.5 ± 1.6	4.3 ± 1.7	0.40	3.5 ± 1.6	3.9 ± 2	0.66	3.2 ± 1.1	4.1 ± 2	0.22	3 ± 1.1	3.9 ± 2	0.17

N: Newton, NC: neural component, VC: viscous component, and EC: elastic component.

**Table 3:** Nonparametric correlations in PD subjects between total and neural components of passive movement resistance in hand/finger muscles and clinical ratings of rigidity.

	Total (N)				NC (N)			
	Passive		Dynamic		Passive		Dynamic	
	Least	Most	Least	Most	Least	Most	Least	Most
UPDRS part III	*R* = 0.49	*R* = 0.46	*R* = 0.46	*R* = 0.42	*R* = 0.39	*R* = 0.45	*R* = 0.40	*R* = 0.40
	*P* = 0.01	*P* = 0.02	*P* = 0.02	*P* = 0.04	*P* = 0.06	*P* = 0.02	*P* = 0.04	*P* = 0.05
Rigidity	**R** = 0.59	*R* = 0.17	**R** = 0.78	*R* = 0.52	**R** = 0.53	*R* = 0.16	**R** = 0.68	*R* = 0.44
	**P** = 0.002	*P* = 0.42	**P** < 0.001	*P* = 0.009	**P** = 0.007	*P* = 0.43	**P** < 0.001	*P* = 0.03
Bradykinesia	*R* = 0.50	*R* = 0.48	*R* = 0.39	*R* = 0.46	*R* = 0.44	**R** = 0.61	*R* = 0.35	*R* = 0.49
	*P* = 0.01	*P* = 0.01	*P* = 0.05	*P* = 0.02	*P* = 0.03	**P** < 0.001	*P* = 0.09	*P* = 0.01

UPDRS = unified Parkinson's disease rating scale, part III motor section, total score. Rigidity and bradykinesia scores of corresponding least and most affected arms. Correlation coefficients shown (Spearman's Rank Order Correlation). Significant correlations marked in bold, *P* < 0.008 (Bonferroni correction).
